# Conventional Spinal Chordomas: Investigation of SMARCB1/INI1 Protein Expression, Genetic Alterations in *SMARCB1* Gene, and Clinicopathological Features in 89 Patients

**DOI:** 10.3390/cancers16162808

**Published:** 2024-08-09

**Authors:** Margherita Maioli, Stefania Cocchi, Marco Gambarotti, Stefania Benini, Giovanna Magagnoli, Gabriella Gamberi, Cristiana Griffoni, Alessandro Gasbarrini, Riccardo Ghermandi, Luigi Emanuele Noli, Chiara Alcherigi, Cristina Ferrari, Giuseppe Bianchi, Sofia Asioli, Elettra Pignotti, Alberto Righi

**Affiliations:** 1 Department of Pathology, IRCCS Istituto Ortopedico Rizzoli, 40136 Bologna, Italy; 2 Department of Spine Surgery, IRCCS Istituto Ortopedico Rizzoli, 40136 Bologna, Italy; 3 Experimental Oncology Laboratory, IRCCS Istituto Ortopedico Rizzoli, 40136 Bologna, Italy; 4 Department of Orthopedic Oncology, IRCCS Istituto Ortopedico Rizzoli, 40136 Bologna, Italy; 5 Department of Biomedical and Neuromotor Sciences (DIBINEM), Alma Mater Studiorum—University of Bologna, 40126 Bologna, Italy; 6 IRCCS Istituto delle Scienze Neurologiche di Bologna, 40139 Bologna, Italy

**Keywords:** conventional chordoma, SMARCB1/INI1, *SMARCB1* gene, FISH analysis

## Abstract

**Simple Summary:**

Alterations in the SMARCB1/INI1 expression pattern have been detected in many tumors, including chordomas. We studied a large group of patients with conventional spinal chordomas, and the aims were to assess the differences in the immunohistochemical expression of SMARCB1/INI1 and the underlying alterations in the *SMARCB1* gene and to investigate the correlation between clinicopathological features and patient survival. Partial SMARCB1/INI1 loss was identified in several patients, and this pattern correlated with mobile spine location and inadequate surgical margins. Moreover, mobile spine tumor location and inadequate surgical margins negatively impacted disease-free survival. The complete loss of SMARCB1/INI1 is currently ongoing as a target for molecular therapy; therefore, the partial loss of SMARCB1/INI1 in tumors could also have therapeutic implications.

**Abstract:**

The partial loss of SMARCB1/INI1 expression has recently been reported in skull base conventional chordomas, with possible therapeutic implications. We retrospectively analyzed 89 patients with conventional spinal chordomas to investigate the differences in the immunohistochemical expression of SMARCB1/INI1 and the underlying genetic alterations in the *SMARCB1* gene. Moreover, we assessed the correlation of clinicopathological features (age, gender, tumor size, tumor location, surgical margins, Ki67 labelling index, SMARCB1/INI1 pattern, previous surgery, previous treatment, type of surgery, and the Charlson Comorbidity Index) with patient survival. Our cohort included 51 males and 38 females, with a median age at diagnosis of 61 years. The median tumor size at presentation was 5.9 cm. The 5-year overall survival (OS) and 5-year disease-free survival (DFS) rates were 90.8% and 54.9%, respectively. Partial SMARCB1/INI1 loss was identified in 37 (41.6%) patients with conventional spinal chordomas (27 mosaic and 10 clonal). The most frequent genetic alteration detected was the monoallelic deletion of a portion of the long arm of chromosome 22, which includes the *SMARCB1* gene. Partial loss of SMARCB1/INI1 was correlated with cervical–thoracic–lumbar tumor location (*p* = 0.033) and inadequate surgical margins (*p* = 0.007), possibly due to the high degree of tumor invasiveness in this site. Among all the considered clinicopathological features related to patient survival, only tumor location in the sacrococcygeal region and adequate surgical margins positively impacted DFS. In conclusion, partial SMARCB1/INI1 loss, mostly due to 22q deletion, was detected in a significant number of patients with conventional spinal chordomas and was correlated with mobile spine location and inadequate surgical margins.

## 1. Introduction

Chordomas are rare malignant neoplasms that develop from embryonic remnants of the notochord. They exhibit distinct histotypes (conventional, poorly differentiated, and dedifferentiated) with different clinical behavior [1]. Conventional chordoma accounts for approximately 95% of cases [1,2]. Chordomas are locally destructive tumors characterized by very slow growth, with possible local recurrence and metastases. The 5- and 10-year OS rates are estimated to be 68.4% and 39.2%, respectively, and the 5- and 10-year DFS rates are 80.9% and 60.1%, respectively [3]. The diagnostic hallmark of chordomas is the nuclear expression of the brachyury protein [1,4]. Complete loss of the SMARCB1/INI1 nuclear protein has also been reported as a peculiar feature of poorly differentiated chordoma [3,5,6]. Recently, the partial loss of SMARCB1/INI1 protein expression has been detected in conventional chordomas localized in the skull base [7]. SMARCB1/INI1 is a tumor suppressor encoded by the *SMARCB1* gene (SWI/SNF-related, matrix-associated, actin-dependent regulator of chromatin, subfamily B, member 1), which is located on the long arm of chromosome 22 (22q11.23). This protein is part of the multisubunit ‘SWItch/Sucrose NonFermentable ATP-dependent chromatin remodelling complex’ (SWI/SNF), which regulates different cellular mechanisms, including gene expression and cell proliferation and differentiation [8,9]. Abnormal expression of SMARCB1/INI1 has been detected extensively in different tumor types, and three distinct expression patterns have been identified: complete loss, partial loss, and reduced expression [10,11]. However, the type of abnormal expression pattern and the type of mutation in the *SMARCB1* gene do not always match; in some cases, no DNA or RNA changes are detected [10]. Among tumors with focal expression of SMARCB1/INI1, different types of genetic alterations have been described, the most frequent being the monoallelic deletion of a portion of the long arm of chromosome 22, which includes the *SMARCB1* gene [7,10]. However, several studies have revealed that SMARCB1/INI1-deficient tumors, despite being very different from each other in location and type, generally share an aggressive clinical course with high local recurrence rates and a prognosis that is often poor [11,12,13,14].

From a treatment perspective, chordoma appears to be resistant to common chemotherapy, and clinical studies are currently ongoing to treat some of these forms with new targeted molecules, including tyrosine kinase inhibitors, CDK4 inhibitors, and immunotherapy based on monoclonal antibodies [2,3,15]. Specifically, the complete loss of SMARCB1/INI1 expression is considered a marker for the evaluation of the effectiveness of Enhancer of Zeste Homolog 2 (EZH2) inhibitors (Tazemetostat) [15,16]. The most frequent cytogenetic abnormalities observed in conventional chordomas are monosomy of chromosome 1 and copy number gains of chromosomes 2, 6, and 7 [1,3]. Loss of chromosome 22 and/or genetic alterations in the *SMARCB1* gene seem to be rare [17,18,19]. 

This study aimed to compare SMARCB1/INI1 protein expression patterns in spinal conventional chordomas with genetic alterations detectable in the *SMARCB1* gene by FISH, clinicopathological features, OS, and DFS.

## 2. Materials and Methods

A retrospective study of 89 patients with conventional spinal chordoma diagnosed at the Anatomy and Pathological Histology Unit of the Rizzoli Orthopedic Institute from 2010 to 2019 was carried out. In order to perform morphological, immunohistochemical, and molecular analyses, a formalin-fixed paraffin-embedded (FFPE) tumor tissue sample of adequate size and quality was used, after selection by pathologists (MG and AR). The diagnosis of all the original tumor slides was confirmed independently by two pathologists (MG and AR) via the immunohistochemical expression of brachyury and pan-cytokeratin AE1/AE3. The clinicopathological parameters investigated were: age, gender, tumor size, tumor location, surgical margins, Ki67 labelling index, SMARCB1/INI1 pattern, previous surgery, previous treatment, type of surgery, and comorbidities. The surgical margins were classified according to the Enneking classification [20] and to the Weinstein–Boriani–Biagini (WBB) system [21]. The comorbidities were evaluated by the Charlson Comorbidity Index (CCI) [22]. Ethical committee approval was obtained from the Comitato Etico di Area Vasta Emilia Centro on 27/04/2023 (protocol # CE AVEC: 312/2023/Oss/IOR). As a comparison group, 4 patients with poorly differentiated chordoma were included in the analysis. 

Immunohistochemical staining was performed using an automated immunostainer following the manufacturer’s guidelines (Ventana BenchMark-Ventana Medical Systems, Tucson, AZ, USA), with a mouse monoclonal anti-INI-1 antibody at a concentration of 0.4 μg/mL (MRQ-27; Cell Marque, Rocklin, CA, USA) and a rabbit monoclonal primary anti-Ki-67 antibody at a concentration of 0.2 μg/mL (clone 30-9, Ventana). The immunohistochemical evaluation was executed independently by two pathologists to determine the percentage of proliferating cells (Ki67 labelling index) and to select only samples with partial SMARCB1/INI1 expression and a minimum 10% cut-off of neoplastic nuclei. Regarding SMARCB1/INI1, both patients with mosaic expression (defined by the presence of negative nuclei mixed with positive nuclei) and patients with clonal expression (characterized by the presence of a completely negative high-magnification field alongside a fully positive high-magnification field) were considered eligible; homogeneous nuclear staining in the background of inflammatory cells, stromal fibroblasts, normal epithelial cells, and/or vascular endothelial cells were used as an internal control.

FISH for the *SMARCB1* gene was performed using a commercial SPEC SMARCB1/22q12 Dual color CE/IVD Probe (ZytoVision, Bremerhaven, Germany). The analysis was performed on conventional chordomas with focal SMARCB1/INI1 expression and four poorly differentiated chordomas. The probe included a 545 kb sequence mapped to the 22q11.23 region (ZyGreen fluorochrome label) harboring the *SMARCB1* gene and a 335 kb sequence mapped to the 22q12.1–q12.2 region (ZyOrange fluorochrome label) harboring the *KREMEN1* gene, which was used as an internal control region to detect large chromosome 22q deletions. FISH was performed on interphase nuclei using the Histology FISH accessory kit (Dako, Glostrup, Denmark) according to the manufacturer’s protocol [23], as previously described [7]. For each slide, a minimum of 100 intact nuclei within the tumor area previously marked by the pathologist were scored using a BX41 fluorescence microscope (Olympus, Tokyo, Japan) at 100× magnification, and visible alteration in at least 10% of the cells was considered a positive result. Nuclei with no signal or signals in overlapping nuclei were considered non-informative and were not analyzed. A Color View III CCD camera soft imaging system (Olympus) was used to capture images, which were subsequently analyzed with CytoVision imaging software version 7.5 (Leica Biosystem Richmond Inc., Richmond, IL, USA). The presence of two green signals and two orange signals in a 1:1 ratio was considered the normal copy number pattern; any FISH signals differing from this pattern were classified as altered. The detection of one green signal and one orange signal indicated a monoallelic co-deletion of *SMARCB1* and the control region, which was classified as a monoallelic 22q large deletion, and the presence of additional copies of both green and orange signals indicated a copy number gain (CNG) of chromosome 22.

OS was defined as the time between the date of diagnosis and the date of death or the last follow-up, and DFS was defined as the time between the first disease relapse or metastasis and the last follow-up. Descriptive statistics were used to report patient and clinical characteristics. All the continuous data were expressed as the means and the standard deviations of the means; the categorical data were expressed as frequencies and percentages. Fisher’s chi-square exact test was used to analyze dichotomous variables. Pearson’s chi-square exact test was performed to investigate categorical variables. Kaplan–Meier survival analyses with the log-rank test were performed to assess the influence of the different parameters on OS and DFS. For all the tests, *p* < 0.05 was considered as statistically significant. All the statistical analyses were performed using SPSS v.19.0 (IBM Corp., Armonk, NY, USA).

## 3. Results

Table 1 summarizes the main clinicopathological features of 89 patients with conventional spinal chordomas. 

The dataset included 51 (57.3%) males and 38 (42.7%) females, with a median age at diagnosis of 61 years (range 17–86). Clinically, 43 (48.3%) tumors were located in the cervical–thoracic–lumbar region (mobile spine), while 46 (51.7%) were located in the sacrococcygeal region. The median tumor size at presentation was 5.9 cm (range 1.4–16 cm). The mean CCI of the population was 4.1. Twenty-one patients (23.6%) underwent previous surgical treatment, and 14 patients (15.7%) underwent previous systemic therapy and/or radiotherapy for the same tumor.

Among the 70 patients who underwent surgical resection, 45 patients (50.6%) had adequate surgical margins (wide and radical), while 25 (28.1%) had inadequate surgical margins (intralesional and marginal), according to the Enneking classification [20] (Table S1). Among the remaining 19 inoperable patients, 12 were treated with carbon ion therapy, 3 with proton therapy, and 1 with radiation and chemotherapy; for 3 patients only biopsy information was available without follow-up data. Of the cases with inadequate margins, nine cases were localized at the cervical region, seven cases were localized at the thoracic–lumbar region (six patients were previously treated with surgery at other centers), and nine cases were localized at the sacrococcygeal region (three patients were previously treated with surgery at other centers). When feasible, a classification according to the WBB system [21] was performed and all 10 tumors analyzed had very large extensions with both extra-osseous and intracanal components (Table 2), which did not allow resection with wide margins. 

**Figure 1:**
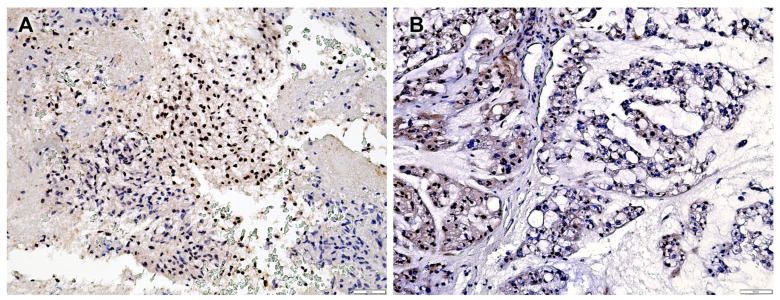
(**A**) Case n.25 showing clonal expression of SMARCB1/INI1; (**B**) case n.44 showing mosaic expression of SMARCB1/INI1.

The median Ki-67 labelling index was 3% (range 1–12%), excluding nine non-evaluable cases (absence of positive internal controls in normal bone marrow cells). The SMARCB1/INI1 immunohistochemical analyses revealed a partial loss of SMARCB1/INI1 (range 10–80%) in 37 (41.6%) patients, while 52 (58.4%) patients exhibited complete protein expression in all neoplastic cells (Table S1). In the 37 patients with focal SMARCB1/INI1 loss, 2 different staining patterns were identified: 27 cases had a mosaic expression pattern (with mixed negative and positive nuclei), while 10 cases had a clonal expression pattern (with separate fully negative and fully positive high-magnification fields) (Figure 1A,B). The four poorly differentiated chordomas exhibited complete loss of SMARCB1/INI1 in all the evaluated neoplastic cells.

Partial loss of the immunohistochemical expression of SMARCB1/INI1 was significantly associated with localization in the cervical–thoracic–lumbar region (*p* = 0.033) and inadequate surgical margins (*p* = 0.007). No significant associations were found with gender, age at diagnosis, tumor size, or Ki67 index (Table 3).

**Figure 2:**
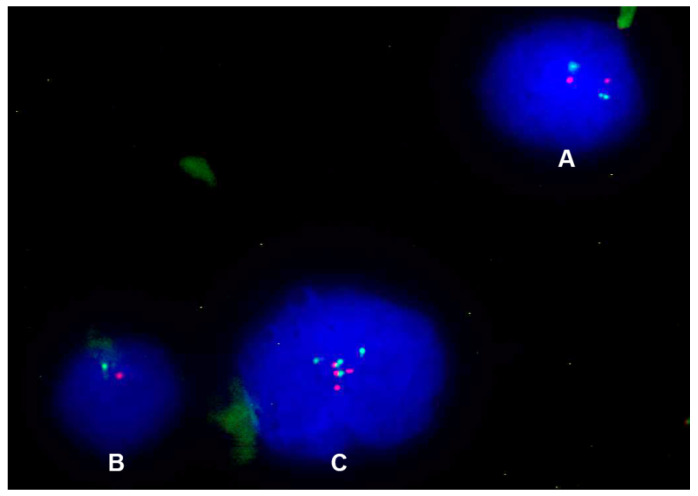
(**A**) Normal nucleus, with two signals for the control region (orange) and two signals for the *SMARCB1* gene (green); (**B**) nucleus with monoallelic deletion, with only one signal for the control region (orange) and only one signal for the *SMARCB1* gene (green); (**C**) nucleus with CNG, with three or more signals for both the control region (orange) and *SMARCB1* gene (green).

The FISH analysis performed on 37 conventional spinal chordoma patients with focal SMARCB1/INI1 loss revealed three possible molecular patterns (Figure 2).

**Figure 3:**
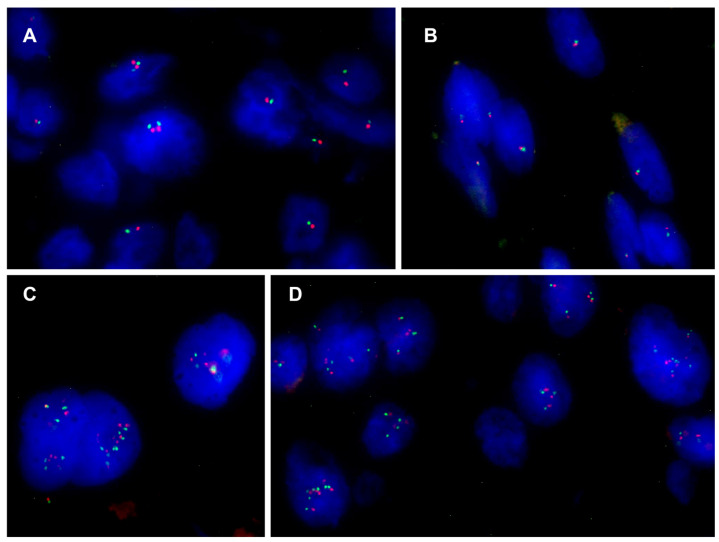
(**A**,**B**) Nuclei with monoallelic co-deletion of the *SMARCB1* gene and the control region from cases n.58 and n.21, respectively; (**C**,**D**) nuclei with CNG from cases n.37 and n.66, respectively.

Monoallelic deletion of the *SMARCB1* gene associated with co-deletion of the control region was observed in 16 cases of conventional chordoma (range 26–94%) (Figure 3A,B); 5 of these also had nuclei with additional copies of both signals (Figure 3C,D). One case exhibited only nuclei with CNG and none with deletions. Due to poor tissue quality, 20 samples did not show hybridized signals and were considered inadequate for FISH scoring (Table S1). Considering the two different staining patterns of focal SMARCB1/INI1 expression, all 10 cases with mosaic patterns had a monoallelic 22q deletion (range 30–94%), 3 of these cases also had nuclei with CNG of both signals; 5 of 6 cases with clonal patterns had a monoallelic 22q deletion (range 26–81%); 2 of these cases also had nuclei with extra copies of *SMARCB1* and the control region, whereas 1 case had only nuclei with CNG of both signals.

**Figure 4:**
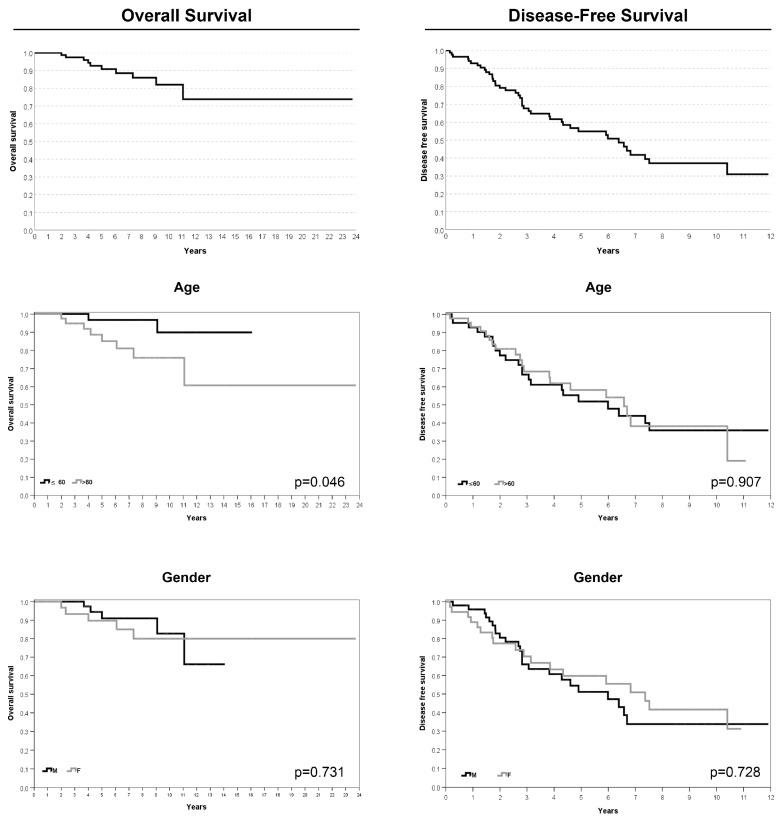
Kaplan–Meier survival analyses for age and gender features.

**Figure 5:**
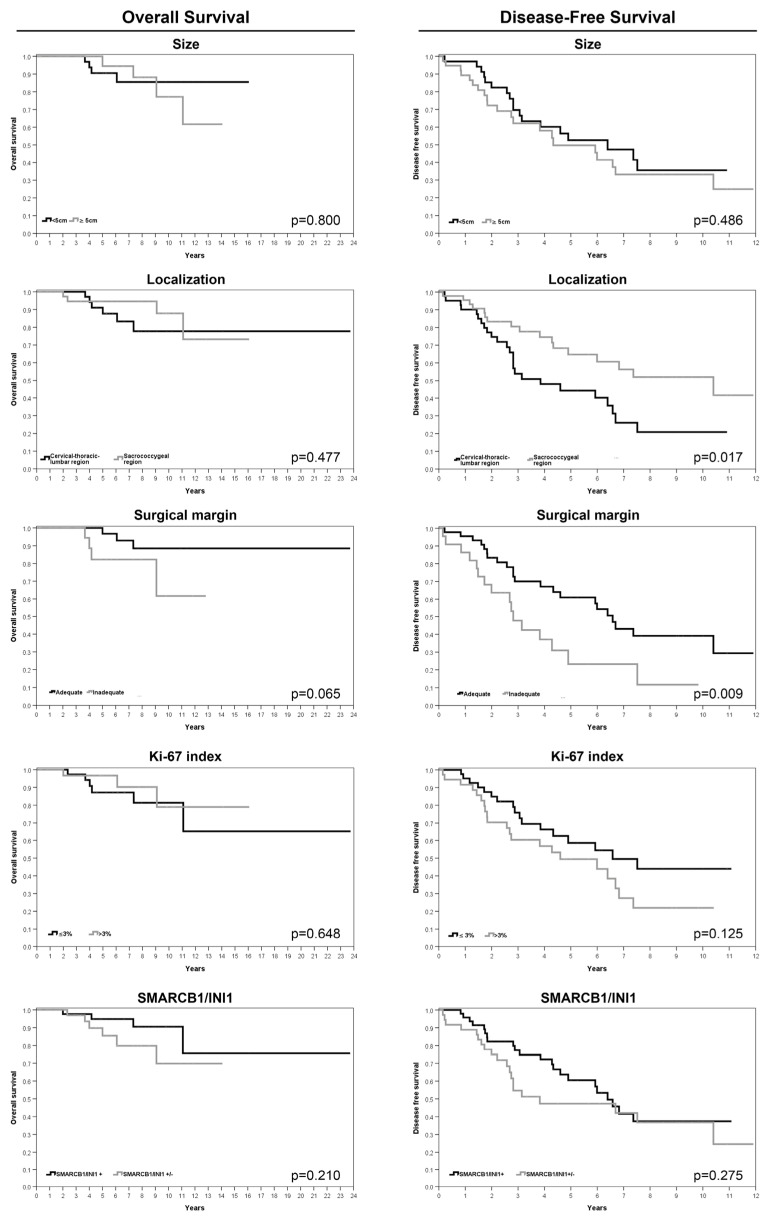
Kaplan–Meier survival analyses for size and tumor localization, surgical margins, Ki-67 index, and SMARCB1/INI1 immunohistochemical expression.

In the four cases of poorly differentiated chordoma, FISH analyses revealed biallelic *SMARCB1* deletions in two cases, a monoallelic deletion in one case, and a pattern with a monoallelic *SMARCB1* deletion associated with an additional control region signal in one case. The average follow-up duration after treatment completion was 66 months (range 2–148). The 5-year OS and 5-year DFS rates were 90.8% (SE 3.6%) and 54.9% (SE 6%), respectively. Univariate analysis revealed worse overall survival for patients older than 60 years (*p* = 0.046). The risk of local recurrence or metastasis was greater for patients with a tumor in the cervical–thoracic–lumbar region (*p* = 0.017), for those with inadequate surgical margins (*p* = 0.009), and for patients who underwent a previous surgery for the same tumor (*p* < 0.0005) (Table 4; Figure 4 and Figure 5). Moreover, the presence of comorbidities significantly affected both OS and DFS, as shown in Table 4 and Table 5.

The results of the multivariate analysis demonstrated that the inadequate surgical margin and an age older than 60 years significantly impaired the OS (Table 6). The risk of local recurrence or metastases was increased by a higher Ki67 index, by an inadequate surgical margin, and by a high CCI: with the same surgical margin and Ki67 scores, the increase of 1 unit of the CCI increases the risk by 40.5% (Table 7). It should be noted that the CCI includes the age, and all patients older than 60 years have a CCI higher than 4.

## 4. Discussion

Conventional spinal chordoma is a rare, slow-growing, locally aggressive malignant neoplasm [1,2]. In recent years, an increasing number of tumors, including poorly differentiated chordomas, have been found to exhibit complete loss of SMARCB1/INI1 protein expression. In many patients, molecular analyses of the *SMARCB1* gene revealed a biallelic deletion [3,11]. Recently, conventional skull base chordomas have also been investigated by immunohistochemistry, and partial loss of SMARCB1/INI1 was identified [7]. In our study, the immunohistochemical pattern of SMARCB1/INI1 in conventional spinal chordomas was analyzed for the first time, and partial loss of SMARCB1/INI1 was observed in 41.6% of cases. In particular, two distinct expression patterns were detected, mosaic and, less frequently, clonal, confirming what has been previously reported on conventional skull base chordomas [7]. From a molecular perspective, several types of genetic alterations have been described among tumors with focal expression, but the most frequent is the monoallelic deletion of a portion of the long arm of chromosome 22 (involving *SMARCB1*) [7,10,16]. However, the genomic studies in the literature revealed that the loss of chromosome 22 or the monoallelic deletion of *SMARCB1* is rare in conventional spinal chordomas [17,18]. In our series, we genetically investigated only conventional chordomas with impaired SMARCB1/INI1 pattern expression, and in 43.2% of the feasible cases, a monoallelic co-deletion of the *SMARCB1* gene and the control region was observed. To evaluate the *SMARCB1* locus at chromosome 22q, we used FISH analysis with a CE-IVD probe. Due to cross-hybridization of chromosome 22 alpha satellites to other centromeric regions, a probe mapped to the 22q12.1-q12.2 region was used as an internal control, which has already been proven to be a reliable control for investigating large deletions [24]. Heterozygous partial deletion of the long arm of chromosome 22 was confirmed as the main molecular mechanism underlying the focal expression of the SMARCB1/INI1 protein. Specifically, the chordomas with mosaic SMARCB1/INI1 expression showed mainly monoallelic 22q deletion, whereas the cases with clonal SMARCB1/INI1 expression were associated with different types of genetic patterns. Nuclei with additional copies of the *SMARCB1* gene and 22q12 control region were also frequently detected in several subclones of cases with deletion, confirming a previously described event [7,16,19]. However, point mutations in *SMARCB1* were not investigated in our study, and epigenetic alterations or post-translational modifications might play an additional role in interpreting the large genetic variability associated with the phenotypic expression of SMARCB1/INI1. We observed that partial loss of SMARCB1/INI1 was significantly associated with the cervical–thoracic–lumbar region (*p* = 0.033) and inadequate surgical margins (*p* = 0.007), suggesting that partial loss of the protein might be associated with increased clinical aggressiveness. A possible reason for the correlation between partial SMARCB1/INI1 loss and inadequate margins could be the major extra-osseous and intracanal involvement of the tumors in the mobile spine, thus increasing the difficulty in obtaining adequate surgical margins. Indeed, 37.5% of patients with inadequate surgical margins were treated for local recurrence of the tumor. The statistical analysis, moreover, indicated the localization in the mobile spine and the presence of surgical inadequate margins as negative prognostic factors in terms of the disease-free survival (*p* = 0.017 and *p* = 0.009, respectively), unlike the cases located in the skull base, where no correlations were found between the partial loss of SMARCB1/INI1 and the clinicopathological parameters evaluated [7]. The multivariate analyses revealed the most crucial factors to be monitored for patient prognosis. The presence of inadequate surgical margins was confirmed as the prevalent risk factor both for OS and DFS; moreover, an age older than 60 years also significantly impaired the OS, whereas DFS was also associated with a high Ki67 index and by a high CCI.

Due to the difficulty in surgically eradicating tumors and the known resistance of chordoma to common chemotherapies [25,26], new molecular targets are being investigated to properly treat these tumors [15]. Increasing knowledge of SMARCB1/INI1 function has enabled the identification of specific targets, including the EZH2 gene. This target is a catalytic subunit of the polycomb repressive complex 2 (PRC2), which plays a role in the chromatin regulation, in cell fate determination, and in cellular differentiation and is often up-regulated in tumors with a loss of SMARCB1/INI1 [8,27,28]. An increase in EZH2 expression correlates with tumor aggressiveness [28], and specifically, this mechanism has been associated with the progression of chordomas [29]. Thus, clinical trials on inhibitors of the EZH2 enzyme are currently underway in tumors with complete loss of SMARCB1/INI1 expression, including poorly differentiated chordomas (ClinicalTrials.gov Identifiers: NCT02601950 and NCT05407441) [30,31,32]. These trials show the safety tolerability and effectiveness of the drug, with the possibility of use in other types of malignancies [2,3,28]; specifically, the potential use of EZH2 inhibitors could also be promising for patients with partial SMARCB1/INI1 loss, but it needs further exploration. 

## 5. Conclusions

In conclusion, we retrospectively analyzed 89 cases of conventional spinal chordoma, and two distinct expression patterns (mosaic and clonal) of partial SMARCB1/INI1 loss were observed. The most frequent molecular alteration detected in conventional chordoma was the monoallelic deletion of the 22q locus (including *SMARCB1* gene). Partial loss of SMARCB1/INI1 was significantly associated with location in the mobile spine and inadequate surgical margins. Inadequate surgical margins, a high Ki67 index, a high CCI, and an age older than 60 years were also associated with a worse prognosis. Treatments with inhibitors of the EZH2 enzyme are currently ongoing in tumors with complete loss of SMARCB1/INI1 expression; therefore, tumors with partial loss of SMARCB1/INI1 could also have therapeutic implications.

## Figures and Tables

**Table 1:** Clinicopathological features of 89 patients with conventional spinal chordomas.

Parameters	All Samples (n = 89)
**Gender (N, %)**	
Male	51 (57.3%)
Female	38 (42.7%)
**Age (median, range in years)**	61 (17–86)
**Age (N, %)**	
≤60 years	42 (47.2%)
>60 years	47 (52.8%)
Tumor size (N, %)	
<5 cm	36 (40.4%)
≥5 cm	39 (43.9%)
Not available	14 (15.7%)
**Tumor localization**	
Cervical–thoracic–lumbar region	43 (48.3%)
Sacrococcygeal region	46 (51.7%)
**Surgical margin**	
Adequate	45 (50.6%)
Inadequate	25 (28.1%)
Not available	19 (21.3%)
**Ki-67 index (median, range)**	3 (1–12)
Ki-67 index (N, %)	
≤3%	43 (48.3%)
>3%	37 (41.6%)
Not evaluable	9 (10.1%)
**SMARCB1/INI1 immunohistochemical expression (N, %)**	
Positive	52 (58.4%)
Positive/negative	37 (41.6%)
**Previous surgery**	
No	53 (59.6%)
Yes	21 (23.6%)
Not available	15 (16.9%)
**Previous treatment**	
No	59 (66.3%)
Yes	14 (15.7%)
Not available	16 (18%)
**Type of surgery**	
En bloc resection	54 (60.7%)
Other surgery	16 (18%)
No surgery	19 (21.3%)
**Charlson Comorbidity Index (CCI)**	
Mean (SD)	4.1 (0.260)

**Table 2:** The WBB classification of patients with surgical inadequate margins.

Case Number	Tumor Localization	WBB Classification	Revision Surgery
1	L3	layers A–E; zones 12–1	NO
2	sacrum	n.a.	NO
7	C4–C5	layers C–E, zones 8–5	NO
15	sacrum	n.a.	NO
19	sacrum	n.a.	NO
25	L5	n.a.	YES
29	C2	layers A–E; zones 11–7	NO
34	C3	layers A–E; zones 2–8	NO
35	C2	layers A–E; zones 9–4	NO
40	L3	n.a.	YES
42	sacrum	n.a.	YES
44	C2–C3	layers A–E; zones 6–2	NO
45	L4–L5	n.a.	YES
48	L2	n.a.	YES
52	sacrum	n.a.	YES
58	T2–T3	n.a.	YES
64	T9	layers A–E; zones 9–1	YES
66	C2	layers A–E; zones 7–4	NO
68	C2	layers A–E; zones 11–5	NO
71	C5–C6	n.a.	YES
72	coccyx	n.a.	NO
73	sacrum	n.a.	YES
78	sacrum	n.a.	NO
79	C1–C2	layers A–E; zones 6–3	NO
89	sacrum	n.a.	NO

n.a. = not applicable, because of localization on the sacrococcygeal region or because of the absence of pre-operative imaging.

**Table 3:** Clinicopathological features according to SMARCB1/INI1 immunohistochemical expression.

	SMARCB1/INI1 + (n = 52)	SMARCB1/INI +/− (n = 37)	*p*-Value
**Gender (N, %)** Male Female			
	28 (53.8%)	23 (62.2%)	0.516
	24 (46.2%)	14 (37.8%)	
**Age (median, range in years)**	61.5 (28–86)	59 (17–79)	0.511
**Age (N, %)**			
≤60 years	22 (42.3%)	20 (54.1%)	0.291
>60 years	30 (57.7%)	17 (45.9%)	
**Tumor size (N, %)**			
<5 cm	20 (38.5%)	16 (43.2%)	
≥5 cm	20 (38.5%)	19 (51.4%)	0.818
Not available	12 (23%)	2 (5.4%)	
**Tumor localization**			
Cervical–thoracic–lumbar region	20 (38.5%)	23 (62.2%)	**0.033**
Sacrococcygeal region	32 (61.5%)	14 (37.8%)	
**Surgical margin**			
Adequate	30 (57.7%)	15 (40.5%)	
Inadequate	8 (15.3%)	17 (46%)	**0.007**
Not available	14 (27%)	5 (13.5%)	
**Ki-67 index** (median, range in percentage)	3 (1–12%)	3 (1–9%)	0.459
**Ki-67 index (N, %)**			
≤3%	26 (50%)	17 (46%)	
>3%	24 (46.2%)	13 (35%)	0.817
Not evaluable	2 (3.8%)	7 (19%)	

Statistically significant *p* values are shown in red color.

**Table 4:** Results from univariate Kaplan–Meier models for OS and DFS.

	5 Years—OS % (SE)	*p*-Value	5 Years—DFS % (SE)	*p*-Value
**Entire sample**	90.8% (3.6%)		54.9% (6%)	
**Gender (N, %)**				
Male	91% (5%)	0.731	51.1% (8.1%)	0.728
Female	89.7% (5.6%)		59.8% (8.8%)	
**Age (N, %)**				
≤60 years	96.8% (3.2%)	**0.046**	51.9% (8.3%)	0.907
>60 years	85.1% (6.2%)		58.3% (8.5%)	
**Tumor size (N, %)**				
<5 cm	90.5% (5.2%)	0.800	52.7% (9%)	0.486
≥5 cm	94.4% (5.4%)		49.7% (9.3%)	
**Tumor localization**				
Cervical–thoracic–lumbar region	87.7% (5.8%)	0.477	44.2% (8.5%)	**0.017**
Sacrococcygeal region	94.6% (3.7%)		64.8% (8.1%)	
**Surgical margin**				
Adequate	96.8% (3.2%)	0.065	61% (8%)	**0.009**
Inadequate	82.2% (9.3%)		23.2% (10.4%)	
**Ki-67 index (N, %)**				
≤3%	89.6% (5.7%)	0.648	60.5% (7.9%)	0.125
>3%	96.7% (3.3%)		47.3% (9.1%)	
**SMARCB1/INI1 immunohistochemical expression (N, %)**				
Positive	94.8% (3.6%)	0.210	58.6% (8.8%)	0.275
Positive/negative	85.5% (6.8%)		49.4% (9.1%)	
**Previous surgery**				
No	88.9% (4.8%)	0.98	66.3% (7.5%)	**<0.0005**
Yes	93.7% (7.4%)		25.3% (10.4%)	
**Previous treatment**				
No	90.6% (4.5%)	0.858	54.4% (7.4%)	0.56
Yes	90.0% (9.5%)		58.4% (14.5%)	
**Type of surgery**				
En bloc resection	88.7% (4.8%)	0.693	61.6% (7.2%)	0.899
Other	90.0% (9.5%)		44.7% (17.1%)	
**Charlson Comorbidity Index (CCI)**				
≤4	92.3%	0.076	63.0% (7.3%)	**0.011**
>4	83.8%		39.3% (10.2%)	

Statistically significant *p* values are shown in red color.

**Table 5:** Univariate analysis for CCI as continuous variable.

**5 years—OS**		***p*-Value**	**HR**	**95.0% CI**	
				**Inferior**	**Superior**
	**CCI**	**0.043**	1.694	1.018	2.820
**5 years—DFS**		***p*-Value**	**HR**	**95.0% CI**	
				**Inferior**	**Superior**
	**CCI**	0.078	1.222	0.978	1.528

Statistically significant *p* values are shown in red color.

**Table 6:** Multivariate analysis for overall survival.

		*p*-Value	HR	95.0% CI	
				Inferior	Superior
**Phase 1**	**CCI**	0.788	1.110	0.519	2.373
	margin (1 vs. 0) *	**0.006**	29.965	2.619	342.854
	**age (˃** **60 vs. ≤60)**	**0.050**	19.600	1.001	383.640
**Phase 2**	margin (1 vs. 0) *	**0.006**	30.049	2.634	342.745
	**age (˃** **60 vs. ≤60)**	**0.012**	24.592	2.019	299.586

* 0 = adequate margin; 1 = inadequate margin. Statistically significant *p* values are shown in red color.

**Table 7:** Multivariate analysis for disease-free survival.

		*p*-Value	HR	95.0% CI	
				Inferior	Superior
**Phase 1**	**Ki67**	**0.037**	1.216	1.012	1.461
	margin (1 vs. 0) *	**0.036**	2.501	1.060	5.904
	**localization**	0.233	0.530	0.187	1.504
	**previous surgery**	0.321	1.489	0.678	3.270
	**CCI**	**0.008**	1.526	1.119	2.079
	**type of surgery (other)**	0.556	0.681	0.189	2.447
	**type of Surgery** **(en bloc resection)**	0.868	0.913	0.313	2.664
**Phase 2**	**Ki67**	**0.033**	1.216	1.016	1.455
	margin (1 vs. 0) *	**0.026**	2.598	1.119	6.032
	**localization**	0.210	0.548	0.214	1.403
	**previous surgery**	0.279	1.529	0.709	3.298
	**CCI**	**0.004**	1.513	1.141	2.007
**Phase 3**	**Ki67**	**0.032**	1.216	1.017	1.453
	margin (1 vs. 0) *	**0.018**	2.771	1.195	6.429
	**localization**	0.203	0.547	0.216	1.383
	**CCI**	**0.004**	1.517	1.143	2.013
**Phase 4**	**Ki67**	0.061	1.188	0.992	1.421
	margin (1 vs. 0) *	**0.019**	2.667	1.173	6.059
	**CCI**	**0.004**	1.502	1.142	1.976

* 0 = adequate margin; 1 = inadequate margin. Statistically significant *p* values are shown in red color.

## Data Availability

The original contributions presented in the study are included in the article. Further inquiries can be directed to the corresponding authors.

## Supplementary Materials

The following supporting information can be downloaded at: https://www.mdpi.com/article/10.3390/cancers16162808/s1.
